# Characterization of superspreaders movement in a bidirectional corridor using a social force model

**DOI:** 10.3389/fpubh.2023.1188732

**Published:** 2023-07-28

**Authors:** Dramane Sam Idris Kanté, Aissam Jebrane, Abdelilah Hakim, Adnane Boukamel

**Affiliations:** ^1^LAMAI, Department of Mathematics, Faculty of Sciences and Technologies, Cadi Ayyad University, Marrakesh, Morocco; ^2^Centrale Casablanca, Complex Systems and Interactions Research Center, Ville Verte, Bouskoura, Morocco

**Keywords:** superspreading events, pedestrian dynamics, contact patterns, social distancing, superspreaders movement, panic, close-contact infections, COVID-19

## Abstract

During infectious disease outbreaks, some infected individuals may spread the disease widely and amplify risks in the community. People whose daily activities bring them in close proximity to many others can unknowingly become *superspreaders*. The use of contact tracking based on social networks, GPS, or mobile tracking data can help to identify superspreaders and break the chain of transmission. We propose a model that aims at providing insight into risk factors of superspreading events. Here, we use a social force model to estimate the superspreading potential of individuals walking in a bidirectional corridor. First, we applied the model to identify parameters that favor exposure to an infectious person in scattered crowds. We find that low walking speed and high body mass both increase the expected number of close exposures. Panic events exacerbate the risks while social distancing reduces both the number and duration of close encounters. Further, in dense crowds, pedestrians interact more and cannot easily maintain the social distance between them. The number of exposures increases with the density of person in the corridor. The study of movements reveals that individuals walking toward the center of the corridor tend to rotate and zigzag more than those walking along the edges, and thus have higher risks of superspreading. The corridor model can be applied to designing risk reduction measures for specific high volume venues, including transit stations, stadiums, and schools.

## 1. Introduction

Superspreaders are infectious individuals who transmit a contagious disease to a larger number of others. Superspreading Events (SSEs) are gatherings where many people are infected at once. They were documented in various infectious diseases such as measles, rubella, monkeypox, smallpox, Ebola hemorrhagic fever, the Severe Acute Respiratory Syndrome (SARS-CoV-1) and COVID-19 (1–5). The definition of a superspreader depends on the pathogen and is often debated even for a single pathogen (6, 7). SSEs largely contribute to the growth of cases, and to counteract this growth public health strategies are adopted. The effectiveness of these strategies depends on people's risk perception and readiness to participate (8–10). This leads to a reduction of production performances and supply chain disruption (11–13). Closure of schools resulted in a learning deficit (14). Closure of services and transition to remote work deteriorated health in certain communities (15–19).

Identifying characteristics, behaviors, and situations that lead to superspreading is critical to slowing transmission and mitigating risks during emerging outbreaks (20–23). Researchers have searched for risk factors using contact tracing and viral genomic data (24). Their analysis have determined a specific viral bottleneck for SARS-CoV-2 transmission, above which individuals can be superspreaders. Some studies suggest high viral loads and high emission of aerosol particles as superspreading factors (25–27). Edwards et al. (28) showed that exhaled virus concentrations tend to increase with COVID-19 infection progression, advanced age, and higher body mass. For diseases that are transmissible via airborne particles or via fomites like SARS-CoV-1 and COVID-19, other factors include virus persistence in the environment due to humidity and temperature, and poor ventilation indoors (29–32).

Mathematical models have also provided insights into the sociological drivers of superspreading. An agent-based model of MERS-CoV transmission (33) conducted a wide monte carlo study and determined the infectiousness of individuals and the number of their contacts as the most critical factors that increase their chances of being a superspreader. Another study devised a susceptible-infectious-recovered (SIR) with spatial structure to elucidate the spatial effects of superspreaders during an epidemic (34). Their analysis suggest that superspreaders are people with many social connections. Another agent-based modeling study suggested that even limiting contacts among people who rarely meet can reduce risks of supersreading (35).

Contact tracing algorithms are extensively used to track the chain of transmission. Kojaku et al. (36) simulations on backward contact tracing on social networks suggest the prevention of a substantial growth in transmissions. Serafino et al. (37) implemented a digital contact tracing over a large Global Positioning System (GPS) data to find the quarantine strategy that breaks the chain with minimal disruption to communities. Mobile phone apps also allow to collect mobility data, however they fail to register contact in some situations (38).

In some cases, we can identify individuals who are likely to have many contacts while they are infectious. For example, children who attend school, adults who have high-contact occupations, or individuals living in congregate housing may have more frequent close encounters than others. In other cases, individuals may become superspreaders through seemingly ordinary daily activities.

Researchers have developed accurate simulations of individual pedestrian dynamics that consider the walker's age, speed, and body weight. Some of these models use cellular automata (CA) to capture the self-organizing patterns that arises within groups of walkers (39, 40). Helbing and Molnar introduced social force models, an alternative framework that uses solid mechanics to describe the movement of pedestrians (41). The movement of each pedestrian is dictated by social and psychological forces. The approach can accurately describe the movement of pedestrians in crowded settings (42–44) and in evacuation situations (45–47). Previous models are not well suited to situations where agents move in different directions and their paths cross or are opposite (48, 49). Smith et al. (50), proposed some modifications where a pedestrian is represented as three overlapping circles. The model allows to simulate avoidance of pedestrians inside concert halls and stadiums. According to Lee et al. (49), lane formation and conflicting pedestrians walking in opposite directions can be modeled using the following effect and the evasive effect. Jiang et al. (51) proposed a dynamic navigation field to describe agent desired direction in bidirectional pedestrian movement. Heliövaara et al. (48) implemented a counterflow model where the area in front of each agent is divided into three overlapping sectors. The counterflow model is formulated as an optimization problem, where each agent lying within a sector either increases or decreases the score of the sector depending on its location and moving velocity. Authors stated that these types of models produce unrealistic trajectories in sparse crowds due to the short range inside which pedestrians react to each other (48). Pedestrians cannot avoid multiple pedestrians simultaneously with these methods especially in dense crowds (52). Wang (53) modified the repulsive forces to account for agents' personal spaces and long-range interactions. Pècol et al. (54–56) have developed a discrete approach to simulate multiple simultaneous collisions. This approach uses pseudo-potentials of dissipation to model local interactions between pedestrians. It was implemented to study congestion (57) and crowd density (44). We will use the framework in Wang (53) and the discrete multiple collision model to simulate movements in sparse crowds, dense crowds, and panic situations. Recently, social force models were applied to estimate infection risks while walking (58) or traveling in an airplane (59), as well as to design optimal mitigation queues (60). We have previously used pedestrian simulation to study the impact of various non-phamarceutical interventions on COVID-19 transmission dynamics in different countries (61–63).

In this work, we use a modified social force model that is calibrated to study the superspreading potential of individuals walking in a bidirectional corridor like those found in transit stations, schools, shopping centers, and office buildings. It is important to note that our work concerns only the case of person-to-person transmission via direct contact. Hence, it is not certain that our findings necessarily apply to infectious diseases transmitted by other routes, such as airborne transmission. We run large numbers of numerical simulations to estimate the distributions of contacts that occur for a pedestrian across a range of conditions, from very low density (social distancing) to very high density (panic). We identify crowding conditions and locations within a corridor that promote superspreading and discuss strategies for mitigating these risks.

## 2. Materials and methods

### 2.1. Movement model in the absence of collisions

**Figure 1:**
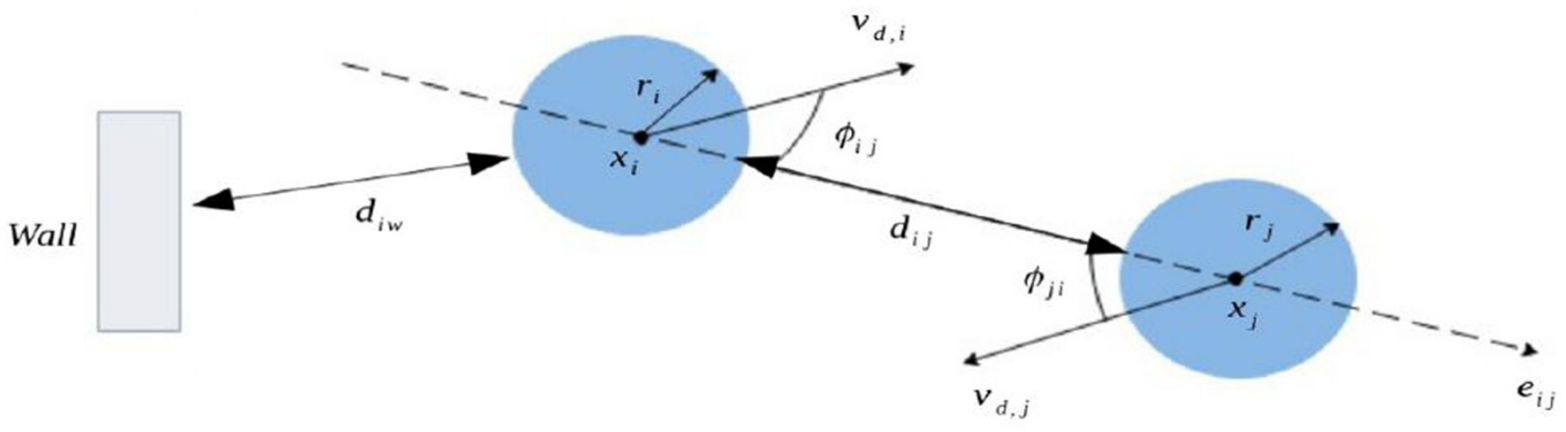
Schematic representation of the model parameters defining the interactions between two pedestrians described as blue disks. Each individual has a center *x*_*i*_ and a radius *r*_*i*_. The distance separating the two individuals *i* and *j* is described by *d*_*ij*_, while we denote the distance separating an individual from a wall *w* by *d*_*iw*_. The desired velocity of each individual is *v*_*d, i*_ and ϕ_*ij*_ is the angle between the direction of the desired velocity of *i* and the distance between the individual *i* and *j*.

In the absence of contacts, regular movement of pedestrians can be described using a social force model (53). The presented microscopic pedestrian movement model has already been introduced in details in previous works (54–56). We use a framework that bring some modifications to the traditional expressions of repulsive forces in Helbing and Molnár (41). It introduces some parameters that make the model consistent, from a psychological point of view, with effects such as time-related stress and interpersonal stress: while moving in public areas, pedestrians tend to keep a certain distance between them. We represent each individual using a disk of center *x*_*i*_ and radius *r*_*i*_. The used notations for model description are graphically represented in Figure 1. We describe the motion of the *i*-th individual using the following equation:


(1) $$\begin{array}{l} m_{i}\frac{d{\text{v}}_{i}}{dt}={\text{f}}_{i}^{self}+{\text{f}}_{i}^{soc}+{\text{f}}_{i}^{obs}, \end{array}$$

where *m*_*i*_ is the mass of the individual, **v**_*i*_ is their velocity, ${\text{f}}_{i}^{self}$ the self-driven force, that describes the adaptation of the pedestrian movement speed to a desired velocity (*v*_*d, i*_). It is given as follows:


(2) $$\begin{array}{l} {\text{f}}_{i}^{self}=m_{i}\frac{{\text{v}}_{d,i}-{\text{v}}_{i}}{\tau_{i}}. \end{array}$$

(3) $$\begin{array}{l} {\text{v}}_{d,i}=v_{d,i}{\text{e}}_{d,i} \end{array}$$

The desired velocity *v*_*d, i*_ is sampled from a normal distribution of mean *v*_*d*_ given in Tables 1, 2, **e**_*d, i*_ is the desired direction, τ_*i*_ is the needed time for the pedestrian velocity to adapt to the desired speed. Next, we introduce the social psychological force exerted by pedestrians toward others:


(4) $$\begin{array}{l} {\text{f}}_{i}^{soc}=\sum \overset{soc}{\underset{ij}{\text{f}}}, \end{array}$$

where ${\text{f}}_{ij}^{soc}$ is the social psychological force between the *i*-th and *j*-th individuals, given as follows:


(5) $$f_{ij}^{soc}=\{\begin{array}{ll} A_{soc}\exp\left(\frac{d_{ij}-d_{soc}}{\beta soc}\right)\left(\gamma +(1-\gamma )\frac{1+\cos\phi_{ij}}{2}\right)e_{ij}, & ifd_{ij}<d_{soc}\\ 0, & elsewhere \end{array}.$$

Here *A*_*soc*_ represents the magnitude of the social psychological force, *d*_*ij*_ is the distance between the two pedestrians *i* and *j*, *d*_*soc*_ describes the distance that individuals tend to keep between them, β_*soc*_ is the falloff length of the social psychological force, while ϕ_*ij*_ represents the angle between the desired velocity and the actual one. After that, we model the interactions between individuals and walls using the force ${\text{f}}_{i}^{obs}$, given as follows:


(6) $$\begin{array}{l} {\text{f}}_{i}^{obs}=\underset{w}{\sum }{\text{f}}_{i}^{w}, \end{array}$$

where ${\text{f}}_{i}^{w}$ is the interaction between the *i*-th individual and the wall *w* described as follows:


(7) $$f_{i}^{w}=\{\begin{array}{ll} A_{obs}\exp\left(\frac{d_{iw}-d_{obs}}{\beta_{obs}}\right)n, & if\;d_{iw}<d_{obs}\\ 0, & elsewhere \end{array}.$$

Here *A*_*obs*_ represents the magnitude of the psychological force between the individual and the wall, *d*_*iw*_ is the distance that separates the individual from the wall, *d*_*obs*_ is the desired distance that each individual aims to keep between from the wall, β_*obs*_ is the falloff length of the psychological force between a person and a wall, and **n** is a normal vector pointing from the wall to pedestrian *i*. We have summarized the parameters used by the model in Table 1.

**Table 1:** A summary of the parameters used in the social force model and the used notations to describe them.

**Parameter**	**Definition**
*v*_*i*_	The real velocity
*v*_*d, i*_	The desired velocity, sampled from a normal distribution
*v*_*d*_	The mean of the desired velocity
τ_*i*_	The needed time to adapt the real velocity to the desired one
*d*_*iw*_	The distance between an pedestrian *i* and the wall *i*
*d*_*ij*_	The distance between the two agents *i* and *j*
*d*_*obs*_	Desired distance an individual aim to keep between them and wall
*d*_*soc*_	Mutual desired distance between agents
*A*_*obs*_	The magnitude of the psychological force between a person and a wall
*A*_*soc*_	The magnitude of the social psychological force
β_*obs*_	Falloff length of the psychological force between a person and a wall
β_*soc*_	Falloff length of the social psychological force
γ	0 < γ < 1, grows with the effect of interactions behind an individual
ϕ_*ij*_	Angle between the desired velocity **v**_*d, i*_ and the vector **e**_*ij*_

**Table 2:** Numerical values of model parameters in scattered crowds in absence of panic.

**Parameter**	**Value**	**Distribution**	**References**
*v*_*d, i*_	*N*(1.34, 0.26) (m/s)	Normal	(41, 64, 65)
*r*_*i*_	[0.15, 0.3] (*m*)	Uniform	(53, 64)
τ_*i*_	[0.15, 0.5] (*s*)	Uniform	(53, 64)
*d*_*obs*_	1.8 (*m*)	–	(53, 66, 67)
*d*_*soc*_	1.8 (*m*)	–	(53, 66, 67)
*A*_*obs*_	10 (*N*)	–	(53, 66, 67)
*A*_*soc*_	10 (*N*)	–	(53, 66, 67)
β_*obs*_	0.8 (*m*)	- -	(53, 66, 67)
β_*soc*_	0.8 (*m*)	–	(53, 66, 67)
γ	(0, 1)	–	(53, 66, 67)

### 2.2. Collison modeling using a non-smooth microscopic approach

We use a non-smooth approach to describe the behavior of individuals during collisions. Let us consider a system of *N* pedestrians represented by circular disks moving in a horizontal plane each defined by a mass *m*_*i*_ an inertia moment *I*_*i*_, a radius *r*_*i*_, a center of gravity *x*_*i*_, whose position with respect to a reference system with axes *x*−*y* and origin *O*, is described by the vector ${}^{t}{\text{q}}_{i}\left(t\right)=\left(q_{i}^{x}\left(t\right),q_{i}^{y}\left(t\right)\right)$ ∈ℝ^2^ and a velocity denoted by ${}^{t}{\text{v}}_{i}\left(t\right)=$ $\left(v_{i}^{x}\left(t\right),v_{i}^{y}\left(t\right)\right)$. For sake of simplicity, the rotation along the *z*-axis has been omitted. The dynamics equations for the set of all pedestrians can be written as follows:


(8) $$\begin{array}{l} \text{M}\left({\text{v}}^{+}-{\text{v}}^{-}\right)=-{\text{p}}^{int}+{\text{p}}^{ext} \end{array}$$

where **M** is the 2*N*×2*N* inertial matrix of the set of individuals; **v**^−^ and **v**^+^ are the pedestrian's velocities before and after the collision. When a contact is detected, the velocities of colliding pedestrians become discontinuous. Therefore, Eq. 8, where the interior and exterior percussions (**p**^*int*^ and **p**^*ext*^ respectively) are introduced, is used to calculate the velocity after the collision. By definition, percussions have the dimension of a linear momentum: a force multiplied by time (kgms^−1^). The **p**^***i**nt*^ percussions are unknown; they take into account the dissipative interactions between the colliding agents (dissipative percussions **p**^*d*^ ) and the reaction forces that permit the avoidance of overlapping among pedestrians (reactive percussions **p**^reac^ ), and hence **p**^*int*^ = **p**^*d*^+**p**^reac^. Frémond (68, 69) defined the deformation velocity $\frac{{\text{Δv}}^{+}{\text{+Δv}}^{-}}{2}$ in duality with **p**^*int*^ according to the work of internal forces, where **Δv** represents the vector containing all the velocities of deformation of individuals in contact. He then introduced a pseudopotential of dissipation Φ, which allows to express **p**^*int*^ as:


(9) $$p^{int}\in \partial \Phi \left(\frac{{\text{Δv}}^{+}+{\text{Δv}}^{-}}{2}\right)$$

where the symbol ∂ denotes the sub differential of the functional in the sense of convex analysis, which generalizes the derivative for convex functions. We recall that a pseudo-potential, in the definition by Moreau, is a non-negative convex function, which is zero for zero dissipation.

### 2.3. Calibration of the model parameters

Several methods were used to identify the values of physical parameters involved in social force models (70–72). For example, some previous works (70, 71) used fundamental diagram to identify these parameters. This diagram assumes a relation between the average walking speed and the density of the crowd. Analytical methods were also used for the determination of parameters. In these methods, parameters were fitted such that the movement characteristics of agents approximate those of pedestrians in organized pedestrian experiments (72) or in real situations (73). The social force model was calibrated to reproduce pedestrian behaviors in normal situations. Johansson et al. (74) estimated model parameters to analyze displacements in urban settings. Wang (53) tuned the parameters to simulate scattered crowds in normal situations. Guo et al. (75) modified the model to simulate pedestrian twice crossing behavior. Heliövaara et al. (48) used the model to study pedestrian counterflow. In this study we use the parameters estimated in Wang (53), Wang and Wang (66), and Trivedi and Pandey (67) to simulate pedestrians in scattered crowds. Values of the parameters are summarized in Table 2. Panic situations also have been extensively studied (53, 76–78). In this study, we use the parameter values determined in a previous study by comparing simulated crowd densities to the ones observed in real settings (44, 57). Panic situations are described through an elevation in the mean desired velocity and the tuning of other parameters, as reported in previous studies (79, 80). The values in Table 2 will be replaced with the estimate in (53, 76–78) to study the impact of panic.

### 2.4. Simulation settings

**Figure 2:**
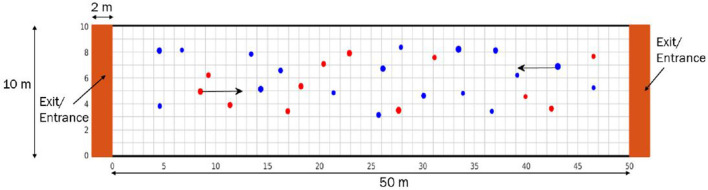
A representation of the domain where numerical simulations are taking place. It corresponds to a bidirectional corridor with two entrances/exits. Red disks represent individuals moving from the left to the right, while blue ones correspond to pedestrians walking from the right to the left. The size of each disk is proportional to the mass of the corresponding individual.

We consider a domain corresponding to a corridor of 50*m*×10*m*. This corridor has two exits which also serve as entrances for pedestrians. At the beginning of the simulation, pedestrians are placed at random locations of the computational domain, and we set their initial velocity to the mean desired velocity. We consider that half of the individuals move from left to right, while the other half move in the opposite direction. A representation of the computational domain is provided in Figure 2. In this figure, red pedestrians move from left to right (**e_i_** = (1, 0)), while blue ones walk in the opposite direction (**e_i_** = (−1, 0)). We consider that simulations last for 10 minutes and 30 seconds. We apply periodic boundary conditions at the two exits to keep the same number of pedestrians during the course of simulations: whenever a pedestrian crosses one of the two exits, they are replaced by another one that has the same mass and speed at a random location on the opposite entrance. We begin our investigation by considering a scattered crowd consisting of 30 individuals, such that the density is equal to 0.06 *p*/*m*^2^. Then, we increase the number of individuals to study the movement of pedestrians in crowded settings. We estimate the characteristics of individuals belonging to two groups: those with a high number of interactions, defined as pedestrians who are responsible for making more contacts than 80 % of the maximal contact number per individual, and those with a low number of interactions, corresponding to pedestrians with a number of contacts lower than 20 % of the maximal contact number per individual in the population.

## 3. Results

### 3.1. Dynamics of potential superspreaders in scattered crowds

In this section, we study the characteristics of individuals with a higher number of contacts who can be potential superspreaders. We consider a small population consisting of 30 pedestrians and investigate the effect compliance to social-distance and walking during panic situations on the prevalence of potential superspreaders.

#### 3.1.1. Potential superspreaders have a high body mass and a lower average walking speed

We begin by applying the model to determine the characteristics of potential superspreaders in a heterogeneous population. To achieve this, we consider a population of pedestrians with randomly sampled weights and means of the desired velocity. We sample the mean of the desired speed for each pedestrian in the range [0.5, 2.3] m/s using a uniform distribution. This situation corresponds to a crowd where some individuals are in a hurry while others walk slower than the average.

We study the relationship between body mass and the chances of being a superspreader. We estimate the body mass of each pedestrian from their radius using the formula $m_{i}=\omega \pi {\left(r_{i}\right)}^{2}$, where ω = 500kg/m^2^ (57). The pedestrian radi are sampled from a uniform distribution such that the body mass of individuals falls between 35.3 and 141.4 kg. Data analysis shows that potential superspreaders have an average body mass of 86 kg, which is 21 % higher than the average body mass of the population (71 kg).

The analysis of the obtained data shows that potential superspreaders have a lower desired velocity on average (1.18 m/s). In comparison, individuals with fewer contacts tend to have a higher desired walking velocity (average *v*_*d*_ is 1.48 m/s).

#### 3.1.2. High compliance with physical distancing prevents superspreading

**Figure 3:**
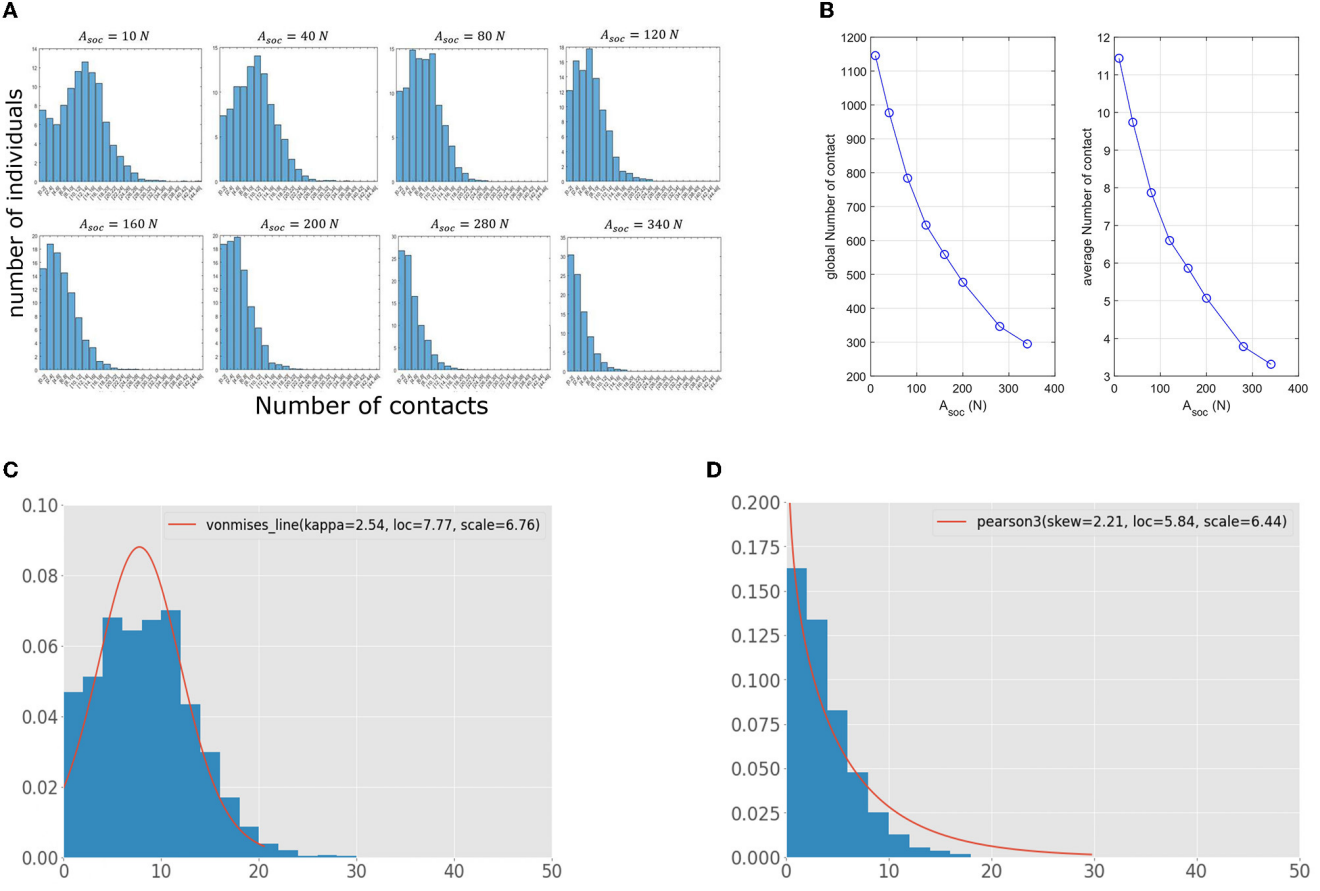
**(A)** Impact of compliance to physical distancing on the distribution of contacts among the pedestrians. The results correspond to the average of 30 numerical simulations for each value. **(B)** The total and average numbers of contacts for different values of *A*_*soc*_. **(C)** Contact distribution for *A*_*soc*_ = 80*N*, fitted to a von Mises distribution. **(D)** Contact distribution for high compliance to social-distancing (*A*_*soc*_ = 340*N*) fitted with a Pearson distribution.

We vary the amplitude of the physical psychological force (*A*_*soc*_) to describe the compliance of individuals to physical distancing. If the value of this amplitude is high, then the individuals tend to keep the desired social distance (*d*_*soc*_) between them. We track the contacts between individuals and calculate the total number of contacts for each individual. We set the average desired velocity for all pedestrians to its normal value *v*_*d*_ = 1.34m/s. We conduct systemic numerical simulations for different values of *A*_*soc*_, and we evaluate the distribution of the total number of contacts for each value (Figure 3A). To minimize the effect of stochastic noises, we consider the average results of 30 simulations of each *A*_*soc*_. The obtained data show a bimodal distribution with two peaks when compliance to physical distancing is very low, with the first peak in zero and the other between 12 and 14 contacts. As we slightly increase compliance with physical distancing, we obtain a unimodal distribution whose peak decreases as we keep increasing the value of *A*_*soc*_. These dynamics suggest that a significant portion of the population could be potential superspreaders if physical distancing is not fully adopted. When adherence to physical distancing is high, most pedestrians would have a low number of contacts. These findings suggest that strong compliance with physical distancing is necessary to prevent superspreading in scattered crowds. Table 3 summarizes the range of contacts made by the highest number of individuals for different values of *A*_*soc*_. It shows that this range decreases from [12, 14] for *A*_*soc*_ = 10*N* to [0, 2] for *A*_*soc*_ = 340*N*. These results demonstrate the important of compliance to physical distancing in not only preventing superspreading, but also reducing the overall risk of disease spread.

**Table 3:** For each value of *A*_*soc*_, we display the interval in which most individuals' number of contacts falls and the corresponding number of individuals.

***A*_*soc*_(*N*)**	**10**	**40**	**80**	**120**	**160**	**200**	**280**	**340**
Range of contacts with highest frequency	[12, 14]	[10, 12]	[4, 6]	[6, 8]	[2, 4]	[4, 6]	[0, 2]	[0, 2]
Average number of individuals in the range	12.6	14.05	14.85	17.75	18.7	19.7	26.7	30.35

This range decreases from [12, 14] for *A*_*soc*_ = 10*N* to [0, 2] for *A*_*soc*_ = 340*N*.

Next, we evaluate the impact of compliance to physical distancing on the contact patterns in the population. Figure 3B shows that both the total and the average numbers of contacts significantly drop when the compliance with physical distancing increases. These findings suggest that the overall risk of disease transmission greatly diminishes when pedestrians abide by physical distancing. To further analyze the effect of *A*_*soc*_ on the distribution of the total number of contacts, we fit the obtained distributions for contacts with the 101 probabilistic distributions available in the stats module of the python library Scipy (81). The best distribution that fits the distribution for low compliance to physical distancing (*A*_*soc*_ = 80*N*) is the von Mises one (Figure 3C), while it is possible to fit data for high compliance level using a Pearson distribution (Figure 3D).

#### 3.1.3. Panic situations increase the relative prevalence of potential superspreaders

Next, we investigate the impact of panic situations on the contact dynamics of the crowds. Panic situations increase the mean desired velocity of pedestrians as shown in a previous work (79). Furthermore, they also change the behavior of pedestrians while walking. To account for these effects, we modify the values of the model as it was done in a previous study (79). In particular, we set the value of *A*_*soc*_ to 2000*N* and we vary the mean of the desired velocity for all pedestrians in the range of [0.5m/s, 2.3m/s]. Other changes introduced to the parameter values are described in Table 4. Changes in the parameter values were introduced to simulate the tendency of individuals to avoid others while walking under panic.

**Table 4:** Numerical values of the parameters used to simulate pedestrian movement under panic and in dense crowds.

**Parameter**	**Value**	**Distribution**	**References**
*r*_*i*_	[0.15, 0.3] (*m*)	Uniform	(44, 53, 64)
τ_*i*_	[0.15, 0.5] (*s*)	Uniform	(44, 53, 64)
*d*_*obs*_	1.8 (*m*)	–	(53, 67)
*d*_*soc*_	1.8 (*m*)	–	(53, 67)
*A*_*obs*_	2, 000 (*N*)	–	(44, 53, 77)
*A*_*soc*_	2, 000 (*N*)	–	(44, 53, 77)
β_*obs*_	0.08 (*m*)	–	(44, 53, 77)
β_*soc*_	0.08 (*m*)	–	(44, 53, 77)
γ	(0, 1)	–	(53, 57)

**Figure 4:**
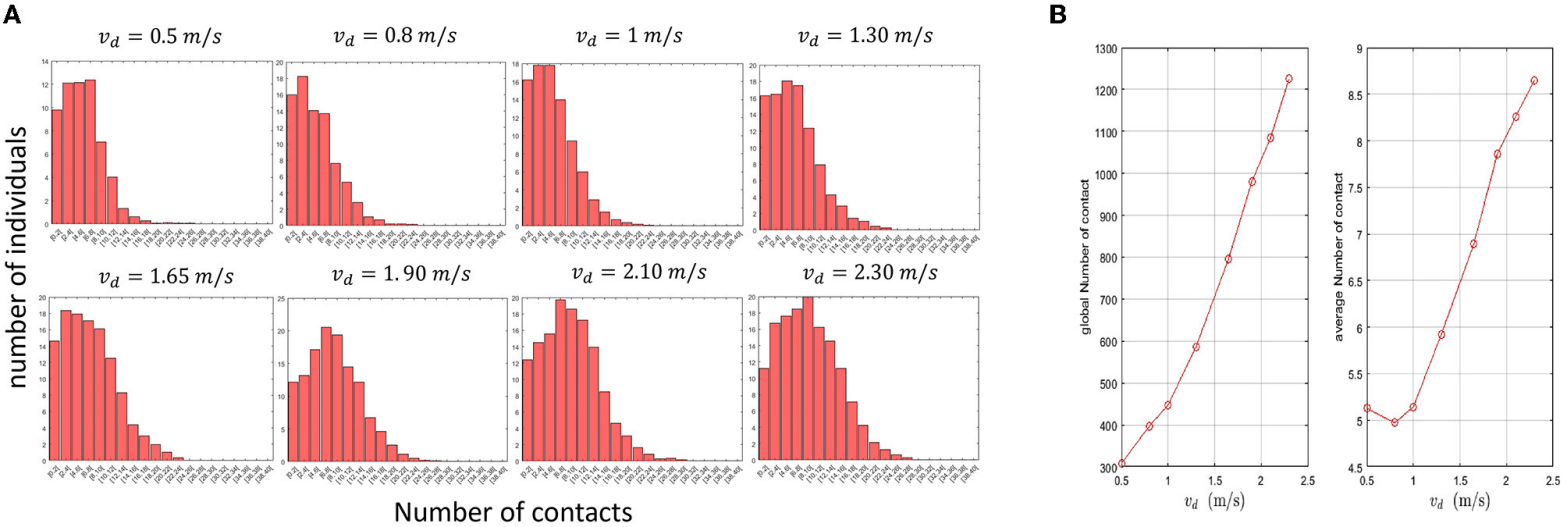
**(A)** Impact of changes in the desired velocity induced by panic situations on the distribution of contacts. **(B)** The total and the average contact numbers in the population for different values of the mean desired velocity (*v*_*d*_).

As before, we track the contacts between pedestrians and analyze the contact distribution in the population. Figure 4A shows the distribution of contacts among pedestrians for different values of the desired velocity. These distributions show that the prevalence of individuals who make a higher number of contacts tend to increase when the mean desired velocity of pedestrians grows. In Figure 4B, we provide the average and the total contact numbers as a function of *v*_*d*_. The obtained results not only confirm that panic situations increase the prevalence of potential supereaders but also elevates the individual risk of getting infected. To further investigate this last point, we determine the ranges of contact that have the highest frequency for different desired velocity means and the corresponding average number of individuals (Table 5). The obtained results show that panic situations increase this range from [2, 8] to [8, 10].

**Table 5:** For each value of *v*_*d*_, we display the interval in which most individuals' number of contacts falls and the corresponding number of individuals.

***v*_*d*_(m/s)**	**0.5**	**0.8**	**1**	**1.30**	**1.65**	**1.90**	**2.10**	**2.30**
Range of contacts with highest frequency	[6, 8]	[2, 4]	[2, 4]	[4, 6]	[2, 4]	[6, 8]	[6, 8]	[8, 10]
Number of individuals in the range	12.35	18.25	17.80	18.05	18.35	20.55	19.7	19.95

Panic situations increase this range from [2, 8] to [8, 10].

#### 3.1.4. Physical distancing reduces the contact duration with superspreaders while panic situations increase it

**Figure 5:**
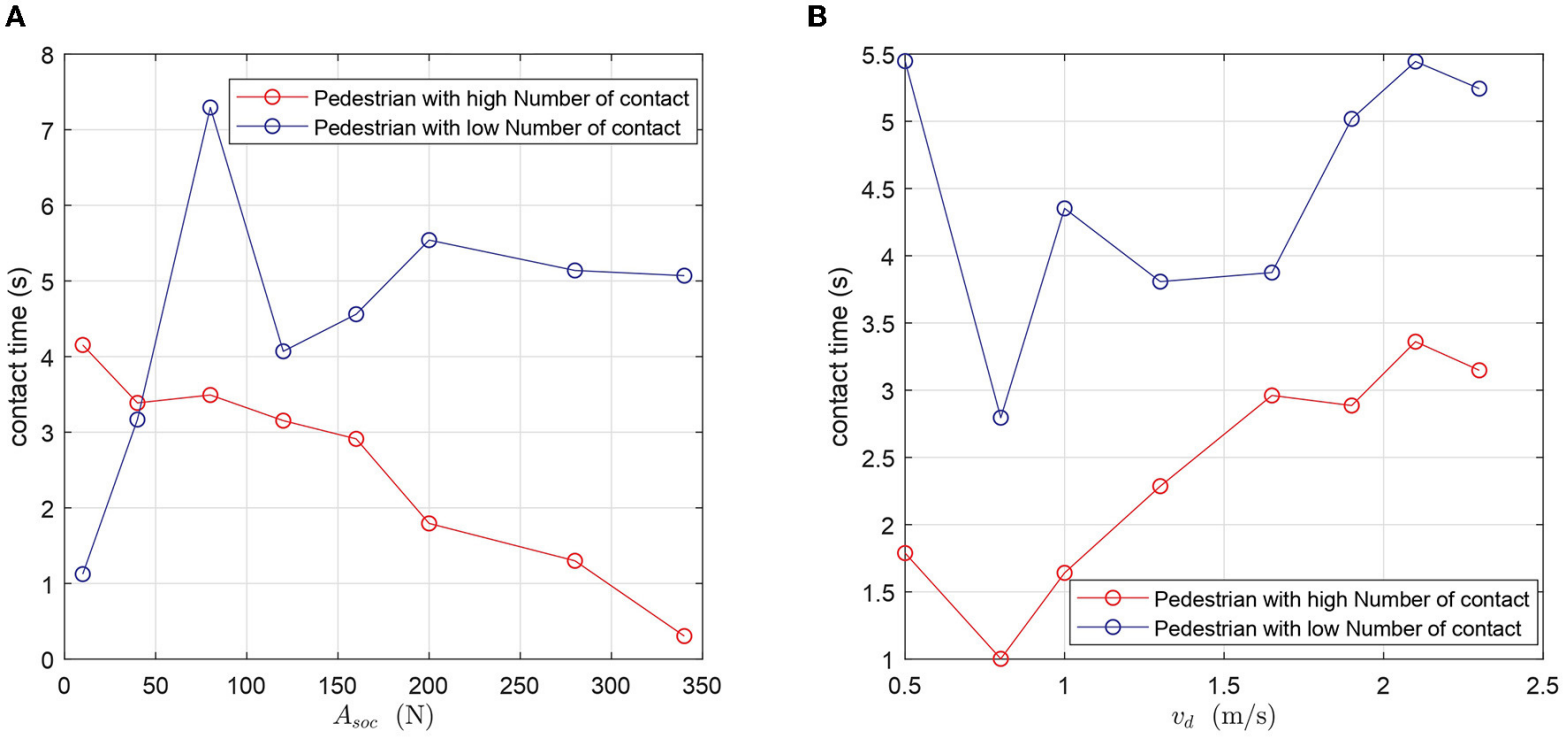
Comparison of the duration of contacts of a pedestrian with a high number of contacts and a pedestrian with a low number of contacts. **(A)** The average duration of contacts as a function of the compliance level to physical distancing. **(B)** The average duration of contacts as a function of the mean desired velocity.

Another aspect that can be explored using our model is the variations in the duration of contacts. Contact duration increases the chances of disease transmission. We study the average contact duration of two population subtypes: individuals who have a high contact frequency, corresponding to those who have more interactions than 80% of the maximal contact number per individual, and individuals with a low contact frequency, i.e., those whose contact number is below 20% of the maximal value. Our analysis reveals that individuals with a higher number of contacts tend to interact with other pedestrians for a shorter time, regardless of their compliance level to physical distancing or the mean desired velocity (Figure 5). The only exception to this rule is when the compliance to physical distancing is very low (*A*_*soc*_ ≤ 40*N*). In this case, pedestrians with a higher contact rate tend to stay in touch with others for a longer time. Another important finding is that compliance to physical distancing not only reduces the relative prevalence of potential superspreaders, but also their average contact duration. In contrast, panic situations increase both their prevalence and their average contact duration.

### 3.2. Movement characteristics of potential superspreaders in dense areas

**Figure 6:**
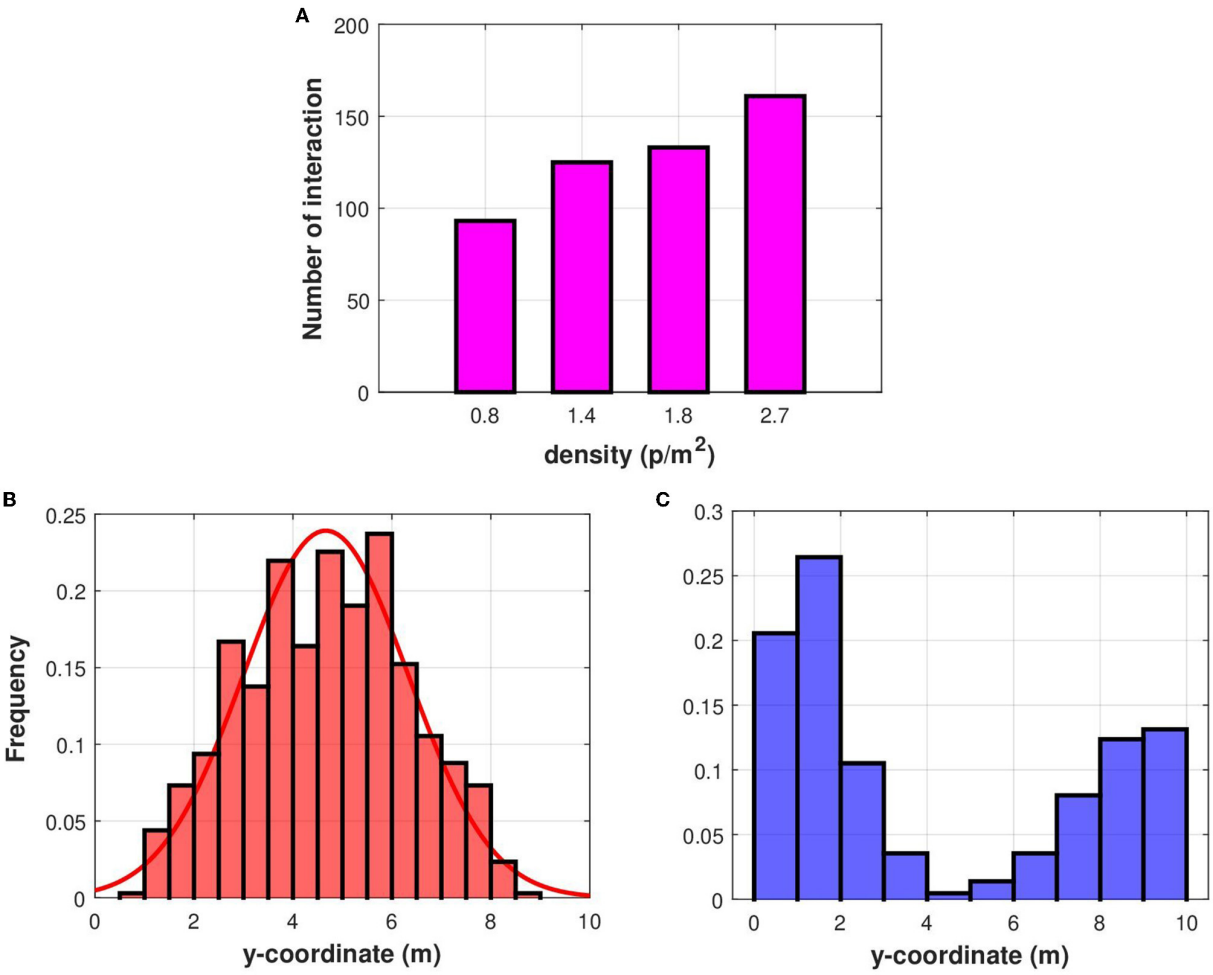
**(A)** A comparison between the average number of contacts for different crowd densities. We also investigate potential superspreaders position in the corridor where the *y*-coordinate varies from 0 to 10 m and the *x*-coordinate varies from 0 to 50 m. **(B)** The distribution of the average *y*-coordinate of potential superspreaders in crowded settings. **(C)** The distribution of the average *y*-coordinate of pedestrians with a lower number of contacts in a dense crowd.

After studying the dynamics of superspreading in scattered crowds, we investigate their characteristics in dense populations. Pedestrians interact more in dense crowds and cannot easily maintain the social distance between them. We quantify the impact of crowd density on the interactions between individuals by varying the number of pedestrians in simulations such that we consider four density values: 0.8 p/m^2^, 1.386 p/m^2^, 1.82 p/m^2^, and 2.72 p/m^2^. Results of numerical simulations suggest that when density grows, the average number of contacts increases, as shown in Figure 6A.

#### 3.2.1. The density of potential superspreaders increases toward the center of the corridor in dense crowds

We continue our investigation by looking at the locations of potential superspreaders. To achieve this, we calculate the average *y*-coordinate for individuals with a high number of interactions. As before, we define potential superspreaders as pedestrians with more contacts than 80 % of the highest contact numbers, while we refer to individuals with low contact numbers as those whose contact number is lower than 20 % of the maximal contact number. The analysis of results from numerical simulations reveals that the concentration of superspreaders increase toward the center of the corridor (Figure 6B), while pedestrians with a low number of contacts can be encountered near the walls (Figure 6C). The frequency of the average *y*-coordinate of superspreaders can be approximated using a normal distribution as shown in Figure 6B. These results suggest that walking near the corridor walls would minimize the overall risk of infection when crowd density is high.

#### 3.2.2. Superspreaders tend to rotate more while walking in crowds

**Figure 7:**
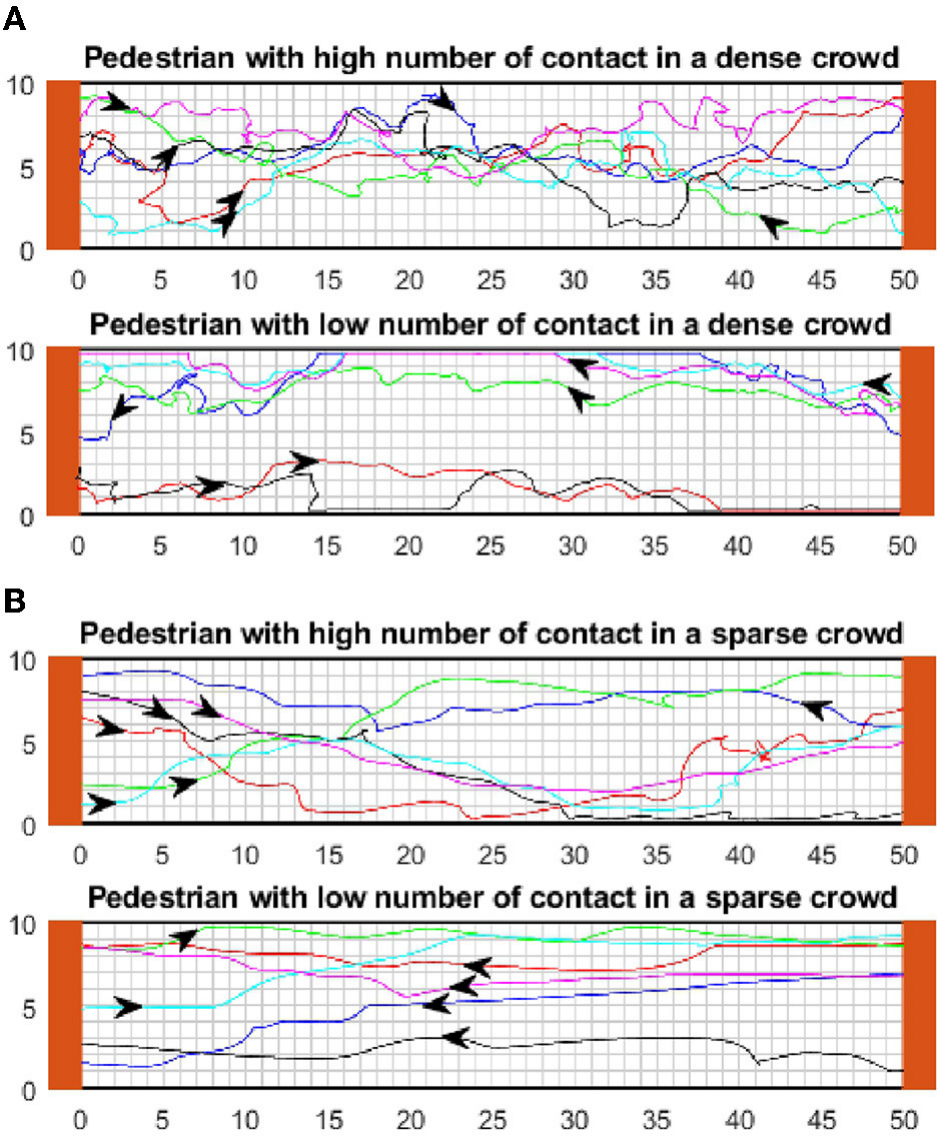
Comparison of trajectories in both dense and scattered crowds. **(A)** Examples of the trajectory of individuals with a high number of contacts. **(B)** Examples of the trajectory of individuals with a low number of contacts.

The trajectories of pedestrians is another feature that can be analyzed using our modeling framework. We compare the movement patterns of potential superspreaders and individuals who have a low number of contacts. A qualitative comparison of the movement trajectories shows that while individuals with a lower contact number walk in a more straight line, those who have a high number of contacts tend to rotate more frequently while walking in crowded areas (Figure 7). These findings apply both in scattered and dense crowds. Thus, potential superspreaders usually walk in zigzag patterns and change the direction of their movement more often. The trajectories of individuals can be used as training data for a classification algorithm that aims to detect potential superspreaders in crowds from real-time images.

## 4. Discussion

Superspreaders play a critical role in promoting and sustaining the spread of infectious diseases (82, 83). As a result, the isolation of superspreaders and the prevention of superspreading can significantly curb the spread of infectious diseases (84). In this work, we have devised a social force model to study the movement characteristics of superspreaders in a bidirectional corridor. One feature of superspreaders is that they tend to have more contacts than average (23, 33, 35). We have focused our present study on these individuals who make a higher number of contacts to be able to interpret the results. Indeed, our findings are more appropriate for diseases that transmit through direct contact with infectious individuals and may not necessarily apply to pathogens that transmit through other routes such as airborne transmission. To study this latter case, we have recently extended the social force framework to include a transmission model that describes the spatial aerosol concentration and the individual risk of infection (85).

The data obtained with our simulations show that potential superspreaders have high body mass and low desired velocities. Since there exist a correlation between advanced age and slower walking speed (86), our results suggest that older people are more prone to be superspreaders. Another previously published study showed an association between severe COVID-19 outcomes and slower walking speed (87). One possible explanation for that is that slower walking people tend to accumulate a higher viral inoculum by getting in contact with more than one infectious individual (88). A prior study identified both advanced age and a higher body mass as risk factors for superspreading, and attributed the finding to elevated concentrations of aerosalized virus in these groups (28). Our analysis suggests an alternative explanation, as both of these characteristics are significantly correlated with elevated contact rates while walking. The social force model can be used to explain this by stating that as mass increases, the magnitude of the repulsive force (or socio-psychological force) weakens and agents are no longer able to maintain a suitable social distance from one another. For COVID-19 and several other viruses, these also happen to be risk factors for severe outcomes (35). During periods of high transmission, precautionary measures such as face masks and social distancing may protect these individuals as well as those around them. We also found that the risk of infection and transmission may correlate with where an individual walks within a corridor. In sparse crowds, superspreaders are distributed evenly across the width of the corridor. In dense crowds, however, they tend to occupy the center of the corridor.

Most of the parameters in social force model have an intuitive physical explanation, which allows us to model sociological phenomena like social distancing and panic events. We find that compliance with physical distancing recommendations reduces both the number and duration of close encounters while walking. This is consistent with prior contact tracing studies (89, 90) and highlights the importance of clear and compelling communication when such measures are warranted, including signage, directional markers, and barriers. Panic events exacerbate risks, increasing the rate of contacts and prevalence of potential superspreaders. Panic may arise during alarming events, in the absence of effective communications and organization. For example, the threat of natural disasters can lead to panic buying, where individuals to rush to stores and form dense crowds in the corridors (91).

We propose an idea to gain insight into superspreading risk factors in a corridor which can be found in transit stations, schools, shopping malls etc. The perspectives of this study are the followings:

- The social force model can be readily calibrated to measure and mitigate superspreading risks in particular crowded settings, such as residences, workplaces, schools, and other public areas.
- The model allowed us to simulate pedestrian movements and interactions, however there are variations of the social force model that account for waiting pedestrians (92) as seen in train stations, and group behavior (93, 94). Other variations could also be used to estimate the risk of infection and study superspreading in specific evacuation scenarios (94).
- Moreover, this method can also be used to estimate contact patterns in different social contexts. It allows to estimate the effect of social distancing, which is lacking in traditional contact matrix estimation methods.

## 5. Conclusion

Based on the social force model, this article proposes a new method for preventing superspreading events and applies the method to study the different factors favoring these events in a corridor. The results show that characteristics such as low walking speed and high body mass increase an individual's risk of exposure. In addition, situations like panic and dense crowds favor superspreading events. The analysis of the movements in the corridor show that the risk of superspreading is much greater in the center of the corridor than along the edges. Finally, we note several limitations of the analysis. Although social force model accurately captures pedestrian movement in dense crowds, it has not yet been validated for crowds across multiple parameter spaces for movement characteristics. The validation of social force models is usually done through comparison with specific settings, where the movement speed and social psychological force are pre-determined. Further, one study suggests that social force models may be unrealistic for some pedestrians when crowd density is varied (95). Another issue is that people do not always behave according to the assumptions of social force models (96). For example, some individuals tend to be unsure about their movement trajectories while others seem to be in a rush. However, we sought to roughly estimate risks of superspreading and identify potential risk factors rather than accurately predict the movement of individual pedestrians. The presented framework can help prevent superspreading during high mass gatherings in different social contexts.

## Data availability statement

The raw data supporting the conclusions of this article will be made available by the authors, without undue reservation.

## Author contributions

DK, AJ, and AH contributed to conception and design of the study. DK and AJ organized the database and implemented the computer code. DK wrote the first draft of the manuscript. AJ, AH, and AB revised the manuscript. AB supervised the project. All authors have read and approved the submitted version.
